# The epidemiology of sexually transmitted co-infections in HIV-positive and HIV-negative African-Caribbean women in Toronto

**DOI:** 10.1186/1471-2334-13-550

**Published:** 2013-11-17

**Authors:** Robert S Remis, Juan Liu, Mona Loutfy, Wangari Tharao, Anuradha Rebbapragada, Stephen J Perusini, Lisungu Chieza, Megan Saunders, LoriAnn Green-Walker, Rupert Kaul

**Affiliations:** 1 Dalla Lana School of Public Health, University of Toronto, Toronto, Ontario, Canada; 2 Women’s College Research Institute, Women’s College Hospital, University of Toronto, Toronto, Ontario, Canada; 3 Women’s Health in Women’s Hands Community Health Centre, Toronto, Ontario, Canada; 4 Public Health Laboratory – Toronto, Public Health Ontario, Toronto, Ontario, Canada; 5 Department of Laboratory Medicine and Pathobiology, University of Toronto, Toronto, Ontario, Canada; 6 Department of Medicine, University Health Network, University of Toronto, Toronto, Ontario, Canada

**Keywords:** Sexually transmitted infections, HIV, Epidemiology, African-Caribbean women, Toronto

## Abstract

**Background:**

HIV disproportionately affects African-Caribbean women in Canada but the frequency and distribution of sexually transmitted infections in this community have not been previously studied.

**Methods:**

We recruited women based on HIV status through a Toronto community health centre. Participants completed a socio-behavioural questionnaire using Audio Computer Assisted Self-Interview (ACASI) and provided blood for syphilis, HIV, hepatitis B and C, herpes simplex virus type 1 (HSV-1), herpes simplex virus type 2 (HSV-2), and human cytomegalovirus (CMV) serology, urine for chlamydia and gonorrhea molecular testing and vaginal secretions for bacterial vaginosis (BV) and human papillomavirus (HPV). Differences in prevalence were assessed for statistical significance using chi-square.

**Results:**

We recruited 126 HIV-positive and 291 HIV-negative women, with a median age of 40 and 31 years, respectively (p < 0.001). Active HBV infection and lifetime exposure to HBV infection were more common in HIV-positive women (4.8% vs. 0.34%, p = 0.004; and 47.6% vs. 21.2%, p < 0.0001), as was a self-reported history of HBV vaccination (66.1% vs. 44.0%, p = 0.0001). Classical STIs were rare in both groups; BV prevalence was low and did not vary by HIV status. HSV-2 infection was markedly more frequent in HIV-positive (86.3%) than HIV-negative (46.6%) women (p < 0.0001). Vaginal HPV infection was also more common in HIV-positive than in HIV-negative women (50.8% vs. 22.6%, p < 0.0001) as was infection with high-risk oncogenic HPV types (48.4% vs. 17.3%, p < 0.0001).

**Conclusions:**

Classical STIs were infrequent in this clinic-based population of African-Caribbean women in Toronto. However, HSV-2 prevalence was higher than that reported in previous studies in the general Canadian population and was strongly associated with HIV infection, as was infection with hepatitis B and HPV.

## Background

In 2011, 34 million people worldwide were infected with HIV and 1.7 million died [1]. Most HIV transmission is sexual and it is increasingly clear that common sexually transmitted bacterial and viral co-infections contribute significantly to HIV transmission risk and disease progression [2]. Infections that appear to enhance susceptibility and infectivity through sexual transmission include herpes simplex virus type 2 (HSV-2), human papilloma virus (HPV), syphilis, gonorrhea, vaginal candidiasis, genital chlamydia and bacterial vaginosis (BV) [2-4]. HSV-2, human cytomegalovirus (CMV) and viral hepatitis B and C have been linked to more rapid disease progression and increased mortality [5-8]. Therefore, a better understanding of the epidemiology of these co-infections in HIV-affected communities is important.

Sub-Saharan Africa has a disproportionately high prevalence of both HIV and several genital co-infections that have been associated with increased HIV transmission, most notably HSV-2 and HPV [9-12]. Furthermore, African and Caribbean communities in North America and Europe have also been disproportionately affected by HIV [3], and HSV-2 infection is also very common [13].

HIV infection is frequent in African and Caribbean women in Ontario [14], with a rate 24-fold greater than other women [3]. Therefore, we aimed to characterize the epidemiology of HIV and other sexually transmitted infections (STIs) in African-Caribbean (AC) women in Toronto and to apply this knowledge to support community and public health interventions.

## Methods

### Participants and recruitment

The study population consisted of women 16 years of age or older from Africa and the Caribbean living in Greater Toronto. Women were eligible if they self-identified as African or Caribbean and if they, a parent or grandparent were born in sub-Saharan Africa or the Caribbean. We recruited women attending the Women’s Health in Women’s Hands Community Health Centre for medical care or on-site support groups from April 2009 to October 2010. We also recruited some participants from affiliated women’s shelters. Since we aimed to assess the possible associations of co-infections with HIV infection, we recruited both HIV-positive and HIV-negative attendees, with over-sampling of the former. We aimed therefore to recruit 300 HIV-negative and 300-HIV-positive women. The sample size was selected to maximize precision of STI prevalence estimates and for feasibility considerations. The study protocol was approved by the Health Research Ethics Board of the University of Toronto.

Participants completed a self-administered questionnaire using ACASI (Audio Computer Assisted Self-Interview), (Questionnaire Development System (QDS) Version 2.5, Nova Research Company, Bethesda, Maryland, USA) and provided biologic specimens. The questionnaire included demographic information, sexual behaviour (in the previous six months and lifetime, number of sexual partners and condom usage), history of STIs and other medical conditions.

### Sample collection

After informed consent, we collected 20 ml of blood by venipuncture. Participants provided a first-void urine specimen and self-collected vaginal swab for HPV molecular diagnostics. For this purpose, participants inserted the swab into the vaginal vault and inserted it into a collection tube containing transport medium. Vaginal specimens for Gram stain were collected as described by Boskey [15].

### Laboratory methods

HIV testing was performed on serum by enzyme immunoassay (EIA; AxSYM HIV 1/2 gO, Abbott Diagnostics Division, Wiesbaden, Germany). If reactive, the EIA was repeated and confirmed at the Ontario Public Health Laboratory by Ag/Ab testing (Architect Ag/Ab Combo, Abbott Diagnostics, Abbott Park, IL, USA) and by Western blot. Serologic testing was also performed for herpes simplex, types 1 and 2 (Herpes Simplex Virus ELISA IgG, Focus Diagnostics, Cypress, CA, USA); human cytomegalovirus (CMV; AxSYM CMV IgG; Abbott Diagnostics Division, Wiesbaden, Germany); hepatitis B surface antigen (AxSYM HbSAg V2 assay, Abbott Diagnostics); hepatitis B surface and core antibodies (AxSYM Core 2.0, Abbott Diagnostics); hepatitis C virus antibody (HCV, AxSYM HCV Version 3.0), with serologic confirmation of HCV-positive tests (Bio-Rad Monolisa anti-HCV Plus Version 2, Bio-Rad Laboratories, Montreal, Quebec); syphilis rapid plasma reagin (RPR, Pulse Diagnostic Inc, Burlington, ON) and TPPA (Serodia TP.PA, Fujirebo Inc). *Neisseria gonorrhoeae* and *Chlamydia trachomatis* were detected in urine by nucleic acid amplification testing (ProbeTec™ ET Amplified DNA Assay, Becton Dickinson, Franklin Lakes, NJ, USA). Bacterial vaginosis (BV) was diagnosed by the Nugent criteria using the results from a Gram stain smear of the vaginal swab [16]. A score of zero to three was considered normal, four to six was classified as abnormal vaginal flora (or intermediate), and seven to 10 was defined as BV. Trichomonas vaginalis infection was detected with the PCR methodology as described by Caliendo et al. [17]. Prior to applying this method for study samples, the sensitivity of this published PCR assay was verified in-house with a panel of known positive samples and lack of cross reactivity confirmed by testing against a panel of 40 common genito-urinary bacterial species. As found by numerous other studies such as Schewbke et al., molecular testing has greater sensitivity than wet mount for clinically relevant Trichomonas vaginalis detection [18]. Human papilloma virus infection with either 13 high risk (HR) or five low risk (LR) types was detected from the vaginal swab with the HPV HR/LR HC2 test (Digene Corporation, Gaithersberg, MD, USA). The HR/LR HC2 test detects virus above 5,000 copies/ml by measuring a chemiluminescent signal (relative light units, RLU) upon hybridization of probes for LR HPV (6, 11, 42, 43, 44) or HR HPV (16, 18, 31, 33, 35, 39, 45, 51, 52, 56, 58, 59, 68). We calculated the ratio of RLU to establish the cutoff: specimens with 1.0 or greater were considered positive and below 1.0 negative. Participants were considered actively infected by hepatitis B if HBsAg was present and ever infected if HBsAg, anti-HBc or anti-HBs (if not vaccinated) were detected.

### Statistical analysis

The data from the ACASI questionnaire and the laboratory results were analyzed in SAS (Version 9.3, SAS, Cary, NC). We examined the prevalence of STIs (and exact binomial 95% confidence intervals) of co-infections stratified by HIV status. We also examined the correlates of chlamydial genital infection; in univariate analysis, four variables were significantly related to chlamydia. However, quasi-complete separation occurred and maximum likelihood estimates could not be obtained when multivariate logistic regression was performed. Therefore, we used a series of four PROC FREQ procedures with the Cochran-Mantel-Haenszel option. In this approach, each variable was used as the predictor and the remaining three variables were used as stratifying, or controlling, variables.

## Results

### Study population

417 women (126 HIV-positive and 291 HIV-negative) were included in the analysis. Their demographic characteristics are shown in Table 1. HIV-negative women were younger and more likely to be single than HIV-positive women. Also, significantly more HIV-positive women were born in Africa (81.0%) than HIV-negative women (36.4%). The majority (59.5%) of HIV-positive women were refugee claimants, higher than in HIV-negative women (36.4%).

**Table 1:** Demographic characteristics among HIV-positive and HIV-negative African-Caribbean women in Toronto

	**HIV-positive**	**HIV-negative**	**p value**
Total participants	126	291	
Age (years)			
Mean	40.3	33.9	<0.0001^a^
Median (IQR)	40 (34-46)	31 (24-42)	
15-24	2 (1.6%)	78 (26.8%)	
25-34	30 (23.8%)	96 (33.0%)	
35-44	58 (46.0%)	59 (20.3%)	
45+	36 (28.6%)	58 (19.9%)	
Education			
No education	4 (3.2%)	7 (2.4%)	NS
Some/completed elementary school	11 (8.8%)	31 (10.7%)	
Some/completed secondary school	46 (36.8%)	118 (40.8%)	
Some/completed college/university	61 (48.8%)	119 (41.2%)	
Some/completed graduate education	3 (2.4%)	14 (4.8%)	
Marital status			
Married/common-low	22 (18.0%)	72 (25.4%)	<0.0001
Separated/divorced/widowed	57 (46.7%)	49 (17.3%)	
Single	43 (35.2%)	163 (57.4%)	
Annual household income			
Less $10,000	38 (35.2%)	102 (55.1%)	<0.0001
$10,000 - $19,999	37 (34.3%)	29 (15.7%)	
$20,000 - $49,999	30 (27.8%)	34 (18.4%)	
$50,000 or more	3 (2.8%)	20 (10.8%)	
Language spoken			
N	118	278	
English	103 (87.3%)	252 (90.3%)	NS
French	15 (12.7%)	32 (11.5%)	NS
Spanish	0 (0.0%)	3 (1.1%)	NS
Other	44 (37.3%)	49 (17.6%)	<0.0001
Region of birth			
Africa	102 (81.0%)	106 (36.4%)	<0.0001^b^
Caribbean	21 (16.7%)	131 (45.0%)	
Canada	2 (1.6%)	50 (17.2%)	
Other	1 (0.8%)	4 (1.4%)	
Immigration status at first arrival in Canada			
Landed/permanent resident	25 (20.7%)	58 (25.1%)	0.0008^b^
Refugee claimant	72 (59.5%)	84 (36.4%)	
Temporary worker	1 (0.8%)	3 (1.3%)	
Visitor	17 (14.0%)	71 (30.7%)	
Student	2 (1.7%)	6 (2.6%)	
Non-status	4 (3.3%)	9 (3.9%)	

^a^Wilcoxon Rank Sum Test.

^b^Fisher’s Exact Test.

### Bacterial infections

The prevalence of bacterial STIs among HIV-positive and HIV-negative women is summarized in Table 2. Chlamydia, gonorrhea and syphilis were infrequent. Only chlamydial infection prevalence differed by HIV status, being higher in HIV-negative women. BV was found in 15.4% of HIV-positive women, similar to the 17.3% among HIV-negative women.

**Table 2:** Prevalence of bacterial and viral pathogens among HIV-positive and HIV-negative African-Caribbean women in Toronto

	**HIV-positive**			**HIV-negative**			**p value**
	**Tested**	**Positive**	**Prevalence % (95% CI**^**a**^**)**	**Tested**	**Positive**	**Prevalence % (95% CI)**	
Chlamydia	123	0	0.0% (0.0-2.9%	286	11	3.8% (1.9-6.8%)	0.039^b^
Gonorrhea	124	0	0.0% (0.0-2.9%)	285	0	0.0% (0.0-1.3%)	NS
Syphilis	126	1	0.79% (0.02-4.3%)	291	1	0.34% (0.01-1.9%)	NS
Bacterial vaginosis	123	19	15.4% (9.6-23.1%)	283	49	17.3% (13.1-22.2%)	NS
Abnormal vaginal flora	123	42	34.1% (25.8-43.2%)	283	84	29.7% (24.4-35.4%)	NS
*Trichomonas vaginalis*	124	9	7.3% (3.4-13.3%)	286	13	4.5% (2.4-7.6%)	NS
Yeast	123	9	7.3% (3.3-13.4%)	283	19	6.7% (4.1-10.3%)	NS
HSV-1	123	111	90.2% (83.6-94.6%)	290	254	87.6% (83.2-91.2%)	NS
HSV-2	124	107	86.3% (79.0-91.8%)	290	135	46.6% (40.7-52.5%)	<0.0001
Cytomegalovirus	126	125	99.2% (95.7-100%)	291	274	94.2% (90.8-96.6%)	0.018
High risk HPV, vaginal	124	60	48.4% (39.3-57.5%	283	49	17.3% (12.9-21.7%	<0.0001
HCV	126	5	4.0% (1.3-9.0%)	290	4	1.4% (0.38-3.5%)	NS
HBV infected^c^	126	6	4.8% (1.8-10.1%)	291	1	0.34% (0.01-1.9%)	0.004^b^
HBV ever^d^	126	60	47.6% (38.7-56.7%)	283	60	21.2% (16.6-26.4%)	<0.0001
HBV vaccination	109	72	66.1% (56.4-74.9%)	248	109	44.0% (37.7-50.4%)	0.0001

^a^95% exact binomial confidence interval.

^b^Fisher’s Exact Test.

^c^Infected with HBV: HBsAg, with or without other HBV markers.

^d^Ever infected with HBV: HBsAg, anti-HBc, or anti-HBs if not vaccinated for HBV.

### Viral infections

The results of serologic testing for viral STIs are also shown in Table 2. All viral pathogens were more prevalent in HIV-positive women and most differences were statistically significant.

Vaginal HPV infection (either HR or LR) was detected in 50.8% of HIV-positive and 22.6% of HIV-negative women (p < 0.0001). Specifically, HR HPV infection was more frequent in HIV-positive women (48.4% vs. 17.3%, p < 0.0001) and the prevalence of LR HPV infection was also higher (26.6% vs. 9.2%, p < 0.0001). HSV-2 infection was markedly more frequent in HIV-positive (86.3%) than HIV-negative (46.6%) women (p < 0.0001).

47.6% of HIV-positive women had serologic evidence of HBV infection versus 21.2% of HIV-negative women. Active HBV infection were also more common in HIV-positive women (4.8% vs. 0.34%, p = 0.004). 66.1% of HIV-positive compared to 44.0% of HIV-negative women reported having been vaccinated against HBV (p = 0.0001). Among those vaccinated in whom the number of doses was known, 58.3% (35/60) HIV-positive and 60.0% (54/90) HIV-negative women received only one or two doses.

We also compared the prevalence of viral infections between African and Caribbean women. While prior HBV infection was more common in African women, both HIV-positive (52.9% vs. 23.8%, p = 0.015) and HIV-negative (33.3% vs. 17.1%, p = 0.004), no significant differences were seen in the other viral infections.

### Covariates of chlamydial infection

The prevalence of chlamydia among HIV-negative women varied by age in years: 15–19, 8.7%; 20–24, 11.5%; 25–29, 3.8%; 30–34, 2.4%; and 35+, 0.0%. The results of the correlation analysis of chlamydia are presented in Table 3. Among HIV-negative women, the prevalence of chlamydia was higher in younger (10.7% in those 15–24 years old compared to 1.4% in those ≥ 25 years old, p = 0.0013) and sexually active women (5.5% in those had had sex in the previous six months compared to 0.0% in those had not, p = 0.015). Chlamydia was more common in women born in Canada (8.3%) than in Africa (1.0%), (p = 0.03). After controlling for other variables indicated in Table 4, chlamydial infection was associated with sex in the previous six months (p = 0.024) and younger age (p = 0.059). After controlling for age, sexual behaviour and region of birth, chlamydia was not significantly associated with HIV status.

**Table 3:** Prevalence of chlamydia and predicting factors among HIV-positive and HIV-negative African-Caribbean women in Toronto

	**HIV-positive**		**HIV-negative**		**Total**	
	**N**	**% chlamydia**	**N**	**% chlamydia**	**N**	**% chlamydia**
	123	0.0%	286	3.8%	409	2.7%
Age (years)						
15-24	2	0.0%	75	10.7%	77	10.4%
≥ 25	121	0.0%	211	1.4%	332	0.90%
p value				0.0013^a^		<0.0001^a^
CMH^b^ controlling for HIV status: p value						0.0014
Had sex in previous 6 months						
Yes	58	0.0%	163	5.5%	221	4.1%
No	62	0.0%	100	0.0%	162	0.0%
p value				0.015^a^		0.012^a^
CMH controlling for HIV status: p value						0.017
Region of birth						
Canada	2	0.0%	48	8.3%	50	8.0%
Caribbean	20	0.0%	129	4.7%	149	4.0%
Africa	100	0.0%	105	1.0%	205	0.5%
Other	1	0.0%	4	0.0%	5	0.0%
p value				0.13^a^		0.012^a^
CMH controlling for HIV status: p value						0.15

^a^Fisher’s Exact Test.

^b^Cochran-Mantel-Haenzel test.

**Table 4:** **Correlates of chlamydial infection, univariate and multivariate**^**a**^

	**Chlamydia-positive**	**Chlamydia-negative**	**Chlamydia-prevalence**	**p value**^**b**^	**Adjusted p value**^**c**^
HIV status					
HIV-positive	0	120	0.0%	0.062	0.25
HIV-negative	9	254	3.4%		
Age (years)					
15-24	6	69	8.0%	0.002	0.059
≥ 25	3	305	0.97%		
Had sex in previous 6 months					
Yes	9	212	4.1%	0.012	0.024
No	0	162	0.0%		
Region of birth					
Canada	4	45	8.2%	0.018	0.48
Caribbean	4	132	2.9%		
Africa	1	192	0.52%		
Other	0	5	0.0%		

^a^Among 383 participants with all data for the variables above.

^b^Fisher’s Exact Test.

^c^Cochran-Mantel-Haenszel controlling other variables in the table.

### Covariates of bacterial vaginosis

BV was not associated with HIV status but was associated with younger age in both HIV-positive and HIV-negative women. Among HIV-positive women, the prevalence of BV varied by age (in years): 15–24, 50.0% (1/2); 25–44, 21.2% (18/67); and 45+, 0.0% (0/36) (p for trend = 0.005). The same trend of decreasing BV prevalence by age was observed among HIV-negative women: 15–24, 27.0%; 25–44, 13.2%; and 45+, 15.5% (p for trend = 0.034). HIV-negative women reporting sex in the previous six months had a higher prevalence of BV (23.5%) than those who did not (10.2%), (p = 0.008).

## Discussion

The African and Caribbean communities in Ontario are disproportionately affected by HIV but, until now, little was known about the prevalence of other STIs which may be important in HIV acquisition and progression. In our study, we recruited women on the basis of HIV status since our goal was not to assess prevalence of HIV, but rather the relationship of co-infections with HIV in this population. We found that classical sexually transmitted infections, i.e. gonorrhea, chlamydia and syphilis, were infrequent. BV was less common than expected and the prevalence did not vary by HIV status. However, persistent (or potentially persistent) viral infections were more frequent in African-Caribbean women in general, particularly in HIV-positive women, with the prevalence of herpes, HPV and CMV being much higher than described in other Canadian populations [19-21]. While our study cannot demonstrate the causal direction of these associations, both HSV-2 and HPV have been found to increase HIV susceptibility [2,4], implying that education and other interventions, including HPV vaccination, might both reduce the high viral STI burden and reduce HIV transmission in these communities. Currently, quadrivalent HPV vaccine (Gardasil) is offered free of charge to Grade 8 students in a school-based public health program in Ontario. Many high-risk types of HPV not infrequent in the population studied here (data not shown) are not included in the present vaccine. Including several of these in an expanded vaccine would be desirable.

HBV infection was prevalent in both HIV-positive and HIV-negative African-Caribbean women though HBV vaccination rates were suboptimal in both groups. HBV vaccine is safe, effective and cost-effective and efforts should be made to reinforce HBV vaccination to protect African-Caribbean women against this serious infection.

Our finding of a low prevalence of most bacterial STI pathogens suggests that they may not play a major role in HIV transmission in the African and Caribbean communities in Toronto. However, given the availability of effective antibiotic treatment for these pathogens and the fact that many of these infections resolve without treatment, the current status of participants may not reflect past infections. Thus, these pathogens could still have played a role in HIV acquisition, especially since HIV-positive women could have been infected before arriving in Canada [22].

Interestingly, we found a significantly higher prevalence of chlamydial infection in HIV-negative compared to HIV-positive women. This was due to younger age and more frequent sex rather than HIV status and we observed no association of chlamydia with HIV positivity after adjusting for age and sexual behaviour [23]. The rate of the chlamydial infection among HIV-negative women was significantly higher among women under 25 years; likely due to both increased sexual activity and the greater biologic susceptibility. This suggests that there is room for more systematic chlamydial screening in this population. At this prevalence, chlamydial screening in this population is probably cost-beneficial [24,25].

The low prevalence of BV was unexpected, as was the lack of association with HIV status. BV has been associated with HIV transmission, both with HIV acquisition in women [26] and with HIV transmission to male partners of HIV-positive women [27]. Furthermore, previous studies have found that women of African and Caribbean descent have an increased prevalence of BV, whether residing in Africa [28] or in North America [29] and that BV is more prevalent in HIV-positive women [30]. In our study, the prevalence of BV in both groups was less than 20%, slightly lower than the 23% observed in white women in the USA, and much lower than the 52% in African-American women in the same study [29]. The reason for the low BV prevalence is not clear, but may relate to the recruitment of women from a clinic-based setting where treatment of common genital infections was available.

While bacterial co-infections were not common, viral STIs were prevalent and strongly associated with HIV status. The prevalence of vaginal HPV (any type) of 22.6% we observed in HIV-negative women was almost double the global average of 11.7% [31] and the 12.7% in Ontario [31], but very similar to the pooled average of 24% reported in women in sub-Saharan Africa [10]. As expected, the prevalence of vaginal HPV infection in HIV-positive women was more than double than that of their HIV-negative counterparts. In particular, we found a strong association of HPV and oncogenic HR HPV with HIV infection status and, among HIV-positive women, the majority of HPV infections were HR strains. While we cannot ascertain whether these HR HPV infections are persistent given the cross-sectional design, our findings reinforce the need for regular cervical cancer screening in this population. Furthermore, we used HybirdCapture2 test to detect HPV DNA present at 5,000 copies or higher. Thus, it is possible that a small subset of individuals might be infected by HPV and yet shed HPV DNA at a level that is below the threshold of detection. The frequency of such an occurrence is unknown. Finally, participants self-sampled for all genital diagnostics, i.e. using either urine or vaginal swabs, and direct cervical samples were not obtained. Vaginal self-sampling is considered a reliable surrogate for clinician collected cervical sampling for HPV DNA detection [32].

We observed a prevalence of HSV-2 among HIV-negative women of just under 50%, very similar to the 48% seen in Black, non-Hispanic women in the US [13] but well above the general population prevalence of 21% or less in women in both the US [13] and Ontario [21]. A recent systematic survey of the health of Canadians carried out in 2009–2011 found a weighted prevalence of HSV-2 of 16.1% in women aged 14 to 59 years [33]. As with previous studies, we found that HSV-2 infection was very strongly associated with HIV infection. It should be noted that the positive predictive value (PPV) of the Focus HSV2 ELISA test may be poor in populations with a low prevalence of HSV-2, at just 38% in a student population with an HSV-2 prevalence of under 4% [34]. However, the PPV was over 93% in a population with a higher HSV-2 prevalence of over 40% [35]. Therefore, we believe that the high HSV-2 prevalence in our participants (48% in the HIV-uninfected women) means that this is less likely to be a concern in our study.

Our cross-sectional study format does not allow us to assess the temporal nature of the relationship between HIV and these viral infections, i.e. whether HPV and HSV-2 infection were acquired before or after HIV infection. It is well established that HSV-2 increases HIV susceptibility [36], while the reverse association is not clear. Furthermore, a strong age association of HSV-2 infection was seen in the HIV-negative participants in our study (data not shown) but this was not apparent in HIV-positive women. Therefore, it is plausible that preceding HSV-2 infection led to enhanced HIV acquisition after subsequent sexual exposure, resulting in a high HSV-2 prevalence in all ages of HIV-positive women. While vaginal HPV infection may also enhance HIV susceptibility [37], HIV infection is known to increase both the incidence and duration of subsequent HPV infection [38], making it harder to hypothesize regarding the temporal nature of this association.

Participants were recruited from a community health centre in downtown Toronto. Thus, subjects may not be representative of African-Caribbean population in Toronto. However, they do represent a broad sample of women by age, region of birth, and education, Furthermore the distribution by these variables is very similar to that of African- and Caribbean-born women in Toronto according to the 2006 Canada Census (data not shown). We were unable to recruit our initial target of 300 HIV-positive women. Nevertheless, the number recruited provided sufficient power to compare these populations for most of the variables of interest. As in all studies of this type, the information on sexual behaviour was self-reported and therefore subject to inaccuracy and potential bias. In general, women may be less likely to report risky sexual behaviours, especially if they are perceived as socially undesirable given the pervasive information promoting condom use.

## Conclusions

Overall, our study found that classical STIs were infrequent in women from the African and Caribbean community in Toronto, Canada, implying that enhanced screening and treatment is not likely to reduce HIV transmission, with the possible exception of chlamydial infection in younger women. However, persistent, or potentially persistent, HSV-2 and HPV viral infections were much more prevalent than in the general population in Ontario, emphasizing the vulnerability of African and Caribbean women to HIV infection and the need to effectively reduce their exposure to HIV.

## Abbreviations

ACASI: Audio computer assisted self-interview; BV: Bacterial vaginosis; CMV: Human cytomegalovirus; QDS: Questionnaire Development System; HPV: Human papillomavirus; HR: High risk; HSV-1: Herpes simplex virus type 1; HSV-2: Herpes simplex virus type 2; LR: Low risk.

## Competing interests

The authors declare that they have no competing interests.

## Authors’ contributions

RSR, RK, ML and WT designed the study. JL analyzed the data. LC recruited the participants and assured the data and sample collection. MS and LAGW supported the study at the clinic and helped to coordinate the subject recruitment. AR and SJP carried out the HPV laboratory analyses. All authors contributed to and approved the final manuscript.

## Pre-publication history

The pre-publication history for this paper can be accessed here:

http://www.biomedcentral.com/1471-2334/13/550/prepub
